# Cross-national measurement invariance of the Purpose in Life Test in seven Latin American countries

**DOI:** 10.3389/fpsyg.2022.974133

**Published:** 2022-09-16

**Authors:** Tomás Caycho-Rodríguez, Lindsey W. Vilca, Mauricio Cervigni, Miguel Gallegos, Pablo Martino, Manuel Calandra, Cesar Armando Rey Anacona, Claudio López-Calle, Rodrigo Moreta-Herrera, Edgardo René Chacón-Andrade, Marlon Elías Lobos-Rivera, Perla del Carpio, Yazmín Quintero, Erika Robles, Macerlo Panza Lombardo, Olivia Gamarra Recalde, Andrés Buschiazzo Figares, Michael White, Carmen Burgos-Videla

**Affiliations:** ^1^Facultad de Ciencias de la Salud, Universidad Privada del Norte, Lima, Peru; ^2^South American Center for Education and Research in Public Health, Universidad Norbert Wiener, Lima, Peru; ^3^Facultad de Psicología, Universidad Nacional de Rosario, Rosario, Argentina; ^4^Centro de Investigación en Neurociencias de Rosario, Facultad de Psicología, Universidad Nacional de Rosario, Rosario, Argentina; ^5^Laboratorio de Cognición y Emoción, Facultad de Psicología, Universidad Nacional de Rosario, Rosario, Argentina; ^6^Consejo Nacional de Investigaciones Científicas y Técnicas, Buenos Aires, Argentina; ^7^Facultad de Ciencias de la Salud, Universidad Católica del Maule, Talca, Chile; ^8^Programa de Pós-Graduação em Psicología, Pontificia Universidade Católica de Minas Gerais, Belo Horizonte, Brasil; ^9^Facultad de Psicología, Universidad Pedagógica y Tecnológica de Colombia, Tunja, Colombia; ^10^Facultad de Psicología, Universidad de Cuenca, Cuenca, Ecuador; ^11^Escuela de Psicología, Pontificia Universidad Católica del Ecuador, Ambato, Ecuador; ^12^Escuela de Psicología, Facultad de Ciencias Sociales, Universidad Tecnológica de El Salvador, San Salvador, El Salvador; ^13^Universidad de Guanajuato, Guanajuato, Mexico; ^14^Facultad de Ciencias del Comportamiento, Universidad Autónoma del Estado de México, Toluca, Mexico; ^15^Facultad de Ciencias de la Salud, Universidad Nacional del Este, Ciudad del Este, Paraguay; ^16^Facultad de Ciencias de la Salud, Universidad Católica de Asunción, Asunción, Paraguay; ^17^Centro de Estudios Adlerianos, Montevideo, Uruguay; ^18^Facultad de Ciencias Humanas y Educación, Universidad Peruana Unión, Lima, Peru; ^19^Instituto de Investigación en Ciencias Sociales y Educación, Universidad de Atacama, Copiapó, Chile

**Keywords:** measurement invariance, cross-cultural, purpose in life, Latin American, Analysis Alignment

## Abstract

The Purpose in Life Test (PIL) is a measure of purpose in life widely used in many cultures and countries; however, cross-cultural assessments are scarce. The present study aimed to evaluate the cross-cultural measurement invariance of the PIL in the general population of seven Latin American countries (Colombia, Ecuador, El Salvador, Mexico, Paraguay, Argentina, and Uruguay). A total of 4306 people participated, selected by non-probabilistic convenience sampling, where Uruguay has the highest mean age (*M* = 41.8; *SD* = 16.6 years); while Ecuador has the lowest mean age (*M* = 24.6; *SD* = 7.8 years). Furthermore, in each country, there is a higher proportion of women (>60%) than men (<40%). Using Multi-Group Confirmatory Factor Analysis, the factorial structure does not show evidence of invariance among the included countries. However, based on the Multi-Group Factor Analysis Alignment, there is evidence that a three-dimensional structure of the PIL (Meaning of existence, Freedom to make meaning in daily life and Will to find meaning in the face of future challenges) is the same in the participating countries. Results based on item response theory indicate that most PIL items can significantly differentiate responses according to the level of life purpose. In addition, people with low life purpose will tend to choose the lower response alternatives on the PIL; while people with higher life purpose will choose higher response alternatives. The findings indicate that the PIL has the potential to increase knowledge about how people conceive and experience their purpose in life in different countries.

## Introduction

The relevance of purpose or meaning in life in the psychological literature increases progressively from the strong connections with positive mental health that research begins to report since 1960 (Yaccarini and Furman, 2017; Bronk, 2020). Within existential philosophy, life is considered to have no single predetermined meaning, as individuals construct meaning throughout their lives (Fabry, 1988; Sezer, 2012). This theoretical idea gained considerable relevance within psychology due to the experience of Victor Frankl, an Austrian physician who suffered confinement in concentration camps during World War II (Furman, 2021; Schimmoeller and Rothhaar, 2021). Frankl believed that humans are driven by a “will to meaning,” which involves the desire to find purpose in life, even in the most miserable circumstances (Frankl, 1984). Following Frankl’s work; Crumbaugh and Maholick (1964) define purpose in life as a subjective sense of one’s life as meaningful. According to the literature, there are three central components in the definition of purpose underlying different conceptualizations: (1) commitment, which refers to the capacity for development, a stable link to certain beliefs, values and orientations, giving a coherent and unified sense of self; (2) goal orientation, which refers to the perception that current activities are related to future outcomes and; (3) personal meaning, defined as the extent or degree of ubiquity of purpose in an individual’s life and its impact on their behavior, thoughts and emotions (Bronk, 2014, 2020). Although the terms “meaning” and “purpose” have been used interchangeably, it has been suggested that there is a differentiation in the use of both terms. Thus, purpose refers to an engagement with the wider world-beyond the self, involving an intention to act for the benefit of others - or, in other words, for a greater cause - something that would differentiate it from merely assigning meaning to one’s own life (Damon et al., 2003; Furman, 2021). However, there is still not enough solid theoretical or empirical evidence to support such a differentiation (Furman, 2021); moreover, Frankl himself, throughout his work, used the terms “purpose” and “meaning” to refer to the same construct (Bronk, 2014).

Lack of meaning or purpose in life is associated with the presence of existential emptiness which, in turn, is related to symptoms of depression, anxiety, stress, addictions or aggressive tendencies (Schulenberg and Melton, 2010). Currently, purpose or meaning in life is a protective factor from the effects of the COVID-19 pandemic on well-being (Arslan and Allen, 2022; Echeverria et al., 2021; Karataş et al., 2021). Despite the changes caused by confinement during the current pandemic, individuals have demonstrated a strong purpose in life (Romero-Ramos et al., 2021), which has been linked to a reduction in symptoms of anxiety, depression, stress, and worry associated with the pandemic (Echeverria et al., 2021; Humphrey and Vari, 2021; Samios et al., 2021; Zuo et al., 2021).

While it is possible to find some exceptions (Ratner et al., 2021; Wang et al., 2021), most research leans toward a quantitative approach to the study of purpose in life (Bronk, 2014, 2020). The first measure was introduced by Frankl (1966) who developed a questionnaire comprised of 13 items, created to assess purpose in a clinical sample. Although Frankl initially developed this scale to provide a clinical assessment, and not for research purposes (Bronk, 2014), Crumbaugh and Maholick (1964) administered their questionnaire in a sample of psychiatric patients and general population. As the results showed reliability and validity below their expectations, with Frankl’s support they developed a new version of the technique (Crumbaugh and Maholick, 1964; Hutzell, 1988). Since its development, the Purpose in Life Test (PIL; Crumbaugh and Maholick, 1964) has been considered the most relevant measure for assessing the degree to which people perceive their own lives as meaningful (Walters and Klein, 1980; Bronk, 2020). Although the PIL is considered to be an eminently quantitative measure, in reality it is composed of Part A, made up of 20 questions, which provide information that can be quantified and compared between different samples; and other parts B, made up of 13 incomplete sentences, and C, which presents paragraphs referring to future goals and past experiences, which provide qualitative information that can be of greater use at the therapeutic level. However, part A is the most used section of the three-part PIL in research studies and can be easily quantified, so this section is frequently referred to as the PIL (Schulenberg and Melton, 2010).

Different versions of the PIL have provided adequate psychometric properties in different contexts such as Australia (Dyck, 1987; Marsh et al., 2003), Argentina (Simkin et al., 2018), Brazil (Hayashi and Esmerelles, 2017; Nascimento and Dias, 2019), Canada (Dufton and Perlman, 1986), Colombia (Ortiz et al., 2012), China (Chang and Dodder, 1983; Law, 2012), Cuba (Ochoa et al., 2018), Korea (Kim et al., 2001), Spain (García-Alandete et al., 2013, 2018), Italy (Brunelli et al., 2012), Hungary (Konkolÿ Thege and Martos, 2006), Japan (Okado, 1998), Mexico (Magaña Valladares et al., 2004), Norway (Haugan and Moksnes, 2013), and Sweden (Jonsén et al., 2010), among others. Despite its wide acceptance within the academic community, one of the main concerns about measuring purpose in life with the PIL lies in the factor structure of the scale. In fact, the PIL has received heavy criticism due to the presence of different underlying factor structures, which limits suggesting a consistently replicable model (Schulenberg and Melton, 2010). Most studies have recommended assessing purpose as a unidimensional concept (Crumbaugh and Maholick, 1964; Marsh et al., 2003; Steger, 2006; Simkin et al., 2018), while others consider a multifactorial structure (García-Alandete et al., 2016).

There are also debates about the number of items that should be included in the scale (García-Alandete et al., 2013). Thus, for example, although the unidimensional structure has been widely replicated (Brunelli et al., 2012; Simkin et al., 2018), other studies have suggested eliminating some items from this model (Marsh et al., 2003; Steger, 2006; Tibaldi Nascimento and Lebre Dias, 2019). Among those who propose a two-dimensional factor structure, there is also a wide disparity of criteria regarding the items and the names of these dimensions (Walters and Klein, 1980; Dufton and Perlman, 1986; Molcar and Stuempfig, 1988; Shek, 1988; McGregor and Little, 1998; Waisberg and Starr, 1999; Morgan and Farsides, 2009; García-Alandete et al., 2013). There is also no consensus among the items and names of the dimensions within those authors who propose a three-factor structure (Magaña Valladares et al., 2004; Jonsén et al., 2010; Martínez Ortiz et al., 2012).

Many of the studies that have suggested a multidimensional structure of PIL indicated the presence of high interfactor correlations (García-Alandete et al., 2013; Hayashi and Esmerelles, 2017). The presence of high correlations has led researchers to test models with a second-order factor underlying all the items (García-Alandete et al., 2013) which can evaluate the existing overlap between the different factors or the degree of independence between them. However, a study with the Spanish version of the PIL indicated that a second-order model was inadequate, due to the presence of incompatible estimates in the standardized equation (García-Alandete et al., 2013). Few studies, such as the one mentioned above, have evaluated second-order or Bifactor models that express general constructs made up of different highly related domains (Chen et al., 2006). Second order models are used when there are highly related dimensions and there is a higher order factor that could explain the relationship between them, while bifactor models, in addition to also assuming the presence of a general factor that would explain the similarity of the dimensions, each item loads on the specific factors and on the general factor, which allows for estimating the proportion of the variance which is a product of the general factor and that which is from the specific factors (Mansolf and Reise, 2017; Raykov and Pohl, 2013). Thus, the second-order and bifactor models would allow for evaluating the effects of the multidimensionality of the PIL on the total score and on each factor (Reise, 2012).

The different results about the factor structure of the PIL exemplify how difficult it is to have measurement instruments with adequate psychometric evidence (García-Alandete et al., 2017). Problems about the structure of the PIL are associated with the use of different factor analysis techniques (exploratory factor analysis, principal components analysis, confirmatory factor analysis) and the different characteristics of the samples (university students, older adults, patients with chronic diseases, among others). Moreover, although the PIL was constructed for clinical purposes, most studies have been conducted on non-clinical samples. Likewise, different cultural and linguistic aspects may influence the factor structure of the scale. When a measurement instrument developed in one cultural context is to be applied in another, it cannot be assumed that the factor structure will be the same (Sharma et al., 2009). This suggests a need for performing a review of different models proposed for the PIL, comparing them and identifying the best and most useful one.

Additionally, culture is an important source of individual values and expectations (Steger et al., 2008), which shapes different experiences that people may consider enriching and meaningful (Kitayama et al., 2000). Some suggest that having a meaningful life is associated with higher levels of well-being within some cultures (Steger et al., 2008); whereas, from well-being theories, it is suggested that, levels of meaning may be consistent across cultures (Ryff and Singer, 1998; Deci and Ryan, 2000). Specifically, if dimensions of meaning in life are associated with basic psychological needs, then these dimensions should manifest and be equally important across cultures. However, other studies suggest cultural variation in life purpose (Lindfors et al., 2006). At the Latin American level, as far as is known from the literature, there are no studies comparing the meaning of life construct among different countries. However, some individual studies have been reported (Francke, 2011; Martínez Ortiz and Castellanos Morales, 2013; Barrero et al., 2020). In Colombia, it has been suggested that additional analysis is needed to identify whether the results are produced by biases in the instrument or the characteristics of the sample (Barrero et al., 2020). Due to the paucity of scientific literature on cultural variation in life purpose, a cross-cultural study is necessary. In these cases, the comparative approach has been useful to identify differences and similarities of a psychological construct between different cultural groups with the aim of making theoretical generalizations. However, as this approach is based on the assumption of comparability; the following question arises: is the same construct truly being compared between different groups? (Lomazzi, 2018). This concern is related to the problem of measurement invariance (MI) and the methodological approaches used to assess it.

Measurement invariance is a procedure that aims to demonstrate to what extent a self-report measure expresses the same meaning and whether the responses to the items are the result of the same factors, in all groups where it was applied (Vandenberg and Lance, 2000). The absence of MI can generate misinterpretations in the results and conclusions possibly derived from methodological errors (Moors, 2004). Traditionally, multigroup confirmatory factor analysis (MGCFA) has been the most widely used method to assess MI (Davidov et al., 2014). This method is based on the concept of “exact equivalence,” where comparisons between different groups will be adequate if the instrument used is exactly the same (Lomazzi, 2018). The MGCFA tests three levels of measurement invariance: configural (where it is evaluated whether or not the construct responds to the same factor structure in all groups), metric (where the unit of measurement needs to be equal, such that factor loadings are equal in all groups) and scalar (which is the most demanding level and requires equality in factor loadings and item intersections) (Steenkamp and Baumgartner, 1998). The presence of metric invariance would allow for the comparison of covariances and unstandardized regression coefficients between groups; whereas, the comparison of latent means would be achievable with the presence of scalar invariance (Davidov, 2010). However, it is also suggested that the presence of partial invariance is sufficient to compare means (Chen, 2007).

Despite the importance of the MGCFA, criticisms of this classical MI approach have emerged for some years (Muthén and Asparouhov, 2013; Van De Schoot et al., 2013; Asparouhov and Muthén, 2014). For example, in large-scale cross-national studies, where a large number of groups can be compared, the MGCFA method may be too stringent and reject models that may be comparable across groups (Lomazzi, 2018). In this sense, it is difficult to achieve full MI as the probability of violating some equivalence principles is higher as the number of groups increases (Davidov et al., 2014). While there are procedures to identify partially invariant models, this requires large modification rates that may lead to produce inadequate models, which are far from the real models and do not guarantee that the means are unbiased; moreover, the selection of the parameters to be released may be arbitrary and miss potentially adequate models (Asparouhov and Muthén, 2014; Marsh et al., 2018).

As an alternative to MGCFA, the Multi-Group Factor Analysis Alignment (CFA-MIAL; Asparouhov and Muthén, 2014) method has been developed and it estimates means without equality restrictions for factor loadings and intersections between groups, thus identifying the most appropriate invariant measurement pattern. In addition, the CFA-MIAL assesses the invariance of factor loadings and intersections simultaneously and considers that both need not necessarily be identical across culturally diverse groups (Asparouhov and Muthén, 2014). The CFA-MIAL assumes that the number of non-invariant parameters and the degree of non-invariance can be minimal. This allows for identifying an invariant pattern between different groups, as well as estimating means and variances by considering the actual differences in factor loadings and intercepts. Thus, CFA-MIAL becomes an alternative which allows for automating and simplifying MI (Marsh et al., 2018). However, CFA-MIAL is still rarely applied (Asparouhov and Muthén, 2014), with only a few relevant studies before (Jang et al., 2017) and during the pandemic (Caycho-Rodríguez et al., 2021a).

While there are some studies that attempt to explore the psychometric properties of the PIL in Latin America (Martínez Ortiz et al., 2012; Simkin et al., 2018) more research evaluating MI across different countries is needed. This could contribute to improved cross-cultural research in the field and provide findings that are more generalizable than previous studies. Therefore, the present study aimed to evaluate cross-cultural MI of PIL in samples from seven Latin American countries (Colombia, Ecuador, El Salvador, Mexico, Paraguay, Argentina, and Uruguay). This included the evaluation of PIL unidimensionality, reliability and cross-cultural MI using the CFA-MIAL and MG-CFA methods. The use of two methods to test MI allows for more solid conclusions and to identify non-invariant items and non-invariant countries. Finally, it is expected that the results will contribute to having a Latin American version of the instrument that can be used in different countries (Yela, 1996).

## Materials and methods

### Design

An instrumental design was used, since the psychometric properties of a psychological measurement instrument were evaluated (Ato et al., 2013).

### Participants

The total sample consisted of 4306 people from seven Latin American countries (Colombia, Ecuador, El Salvador, Mexico, Paraguay, Argentina, and Uruguay), selected by non-probability convenience sampling based on the following inclusion criteria: (1) be of legal age as stipulated in each participating country; (2) be a national of one of the participating countries; (3) be able to answer online surveys and; (4) have given informed consent to participate in the study. Everyone who did not meet the inclusion criteria were not allowed to be part of the study, i.e., people who were minors, who were not nationals of any of the participating countries, who did not have Internet access, and/or who did not give informed consent to participate. Due to movement and interaction restrictions worldwide related to the COVID-19 pandemic during the data collection period, the use of non-probability sampling has been a widely used procedure in the past year (Pierce et al., 2020). Individuals who did not give informed consent to participate were not considered in the study. The highest mean age was found in participants residing in Uruguay (*M* = 41.8; *SD* = 16.6 years); while the lowest mean age was in participants from Ecuador (*M* = 24.6; *SD* = 7.8 years). Furthermore, it can be seen that in all countries there is a higher proportion of women (>60%) than men (<40%). Regarding the educational level of the participants, the majority have completed university studies (>60%). It can also be seen that most of the participants have a professional career (>50%), except in the countries of Ecuador (38.5%) and El Salvador (27.8%), where there is a higher proportion of unskilled work (45.1 and 37.5%, respectively). Table 1 presents the demographic characteristics of the sample for each country.

**TABLE 1:** Sociodemographic characteristics of the participants in the Americas.

Sociodemographic data	Argentina (*n* = 1360)	Colombia (*n* = 317)	Ecuador (*n* = 772)	El Salvador (*n* = 309)	Mexico (*n* = 904)	Paraguay (*n* = 244)	Uruguay (*n* = 400)
Age (M ± SD)	36.4 ± 15.3	32.9 ± 12	24.6 ± 7.8	28.7 ± 8.8	34.6 ± 11.6	36.9 ± 11.5	41.8 ± 12.6
**Gender, *n* (%)**							
Male	284 (20.9%)	81 (25.6%)	273 (35.4%)	91 (29.4%)	267 (29.5%)	48 (19.7%)	100 (25%)
Female	1076 (79.1%)	236 (74.4%)	499 (64.6%)	218 (70.6%)	637 (70.5%)	196 (80.3%)	300 (75%)
**Educational level, *n* (%)**							
Self-taught reading and writing	1 (0.1%)	0 (0%)	3 (0.4%)	1 (0.3%)	1 (0.1%)	0 (0%)	0 (0%)
Basic (<6 years)	0 (0%)	0 (0%)	0 (0%)	0 (0%)	1 (0.1%)	3 (1.2%)	0 (0%)
Primary (≥6 years)	3 (0.2%)	0 (0%)	3 (0.4%)	3 (1%)	1 (0.1%)	0 (0%)	4 (1%)
Secondary (≥9 years)	294 (21.6%)	47 (14.8%)	237 (30.7%)	50 (16.2%)	54 (6%)	21 (8.6%)	56 (14%)
Higher (diploma/bachelor’s degree)	1062 (78.1%)	270 (85.2%)	529 (68.5%)	255 (82.5%)	847 (93.7%)	220 (90.2%)	340 (85%)
**Occupation, *n* (%)**							
Unqualified	271 (19.9%)	59 (18.6%)	348 (45.1%)	116 (37.5%)	123 (13.6%)	31 (21.7%)	23 (5.8%)
Manual Qualified	105 (7.7%)	10 (3.2%)	71 (9.2%)	33 (10.7%)	51 (5.6%)	11 (4.5%)	17 (4.3%)
Qualified non-manual	180 (13.2%)	33 (10.4%)	37 (4.8%)	66 (21.4%)	68 (7.5%)	15 (6.1%)	64 (16%)
Professional	735 (54%)	195 (61.5%)	297 (38.5%)	86 (27.8%)	607 (67.1%)	163 (66.8%)	264 (66%)
Directive	69 (5.1%)	20 (6.3%)	19 (2.5%)	8 (2.6%)	55 (6.1%)	24 (9.8%)	32 (8%)

### Instruments

#### Sociodemographic Survey

Participants completed a sociodemographic survey covering aspects related to age, sex, educational level and occupation.

#### Purpose in Life Test

The PIL assesses meaning in life from a set of 20 items that are answered on a Likert-type scale of seven response categories (Crumbaugh and Maholick, 1964). Categories 1 and 7 have specific response anchors for each item (a score of 1 represents low life purpose and 7 represents high life purpose), and category 4 represents a neutral position. The total score is obtained from the sum of the value of the chosen response on each item, ranging from 20 to 140, where higher scores express clearer meaning and purpose in life. There are no time restrictions, and most respondents answer the 20 questions in 10 to 15 min. The Spanish version by Simkin et al. (2018) was used in this study.

### Procedure

This study is part of a larger project aimed at obtaining brief, cross-culturally valid measures of mental health indicators in Latin America during the COVID-19 pandemic (see, for example, Caycho-Rodríguez et al., 2021a,^2022^, b). The study was conducted between June 12 and September 14, 2020. In all countries, the collection process was the same. A self-administered online survey was prepared on the Google Forms digital platform, which was disseminated through social networks (Facebook, Twitter, WhatsApp, and Instagram) and email. In the self-administered online survey, the objectives of the study, the coordinator’s contact information, and informed consent were presented first. Only after providing informed consent was the self-reported PIL scale accessed. The confidentiality of the data collected was guaranteed; in addition, all participants could withdraw from the study whenever they wished. The project was approved by the Ethics Committee of the Neuroscience Research Center of Rosario and the Laboratory of Cognition and Emotion, belonging to the Faculty of Psychology of the National University of Rosario in Argentina. In addition, the study followed the ethical guidelines of the American Psychological Association (2010).

### Data analysis

Three methodological approaches were used to evaluate the internal structure of the scale: (a) Confirmatory Factor Analysis (CFA), including a Bifactor model, (b) Exploratory Structural Equation Modeling (ESEM) and (c) Bifactor Exploratory Structural Equation Modeling (B-ESEM). All previous psychometric studies of this scale have used classical CFA models (i.e., two- or three-factor related models) to assess the factor structure of the PIL; however, as mentioned above, different studies suggested the presence of multidimensional structures and high interfactor correlations. This may be attributed to the different ways in which people understand life purpose in different cultures or age groups or it might suggest that an adequate analytical model has not been used. In this sense, for multidimensional data, typical CFA models may be too restrictive; whereas, bifactor CFA can give information on multidimensionality due to the coexistence of general and specific factors. CFA models have statistical advantages such as the inclusion of latent variables with measurement error correction (Asparouhov and Muthén, 2009; Marsh et al., 2014). The ESEM model is another viable alternative for a more adequate evaluation of the factorial structure, since it allows cross-loadings (typical of exploratory factor analysis [EFA]) and the use of advanced statistical methods, typical of CFA. Thus, it has been suggested that the ESEM model generates a better fit and fewer correlated factors than the CFA models (Guay et al., 2015). In addition, the ESEM model can be easily and directly compared with previous CFA models. Finally, the B-ESEM model was developed to combine two relevant multidimensionality sources (Bifactor and ESEM) and to have a more accurate assessment of multidimensional measures (Morin et al., 2016). However, when the ESEM model does not present an adequate fit to the data or does not provide smaller interfactor correlations, the CFA model is preferable due to its parsimony (Marsh et al., 2009). Thus, the use of CFA, ESEM and B-ESEM models could contribute significantly to the debate on the structure of the PIL. So far, no study has used all the above-mentioned methods for the evaluation of the factor structure of the PIL.

In the three methodological approaches, the Diagonally Weighted Least Squares with Mean and Variance corrected (WLSMV) estimator was used because the items were at the ordinal level (Brown, 2015). In the CFA models, items were only loaded on their respective factor, while cross-loadings were restricted to zero. In contrast, in the ESEM and B-ESEM models, items loaded on their main factors, while cross-loadings were “targeted,” but not forced, to be as close to zero as possible. For this purpose, a confirmatory rotation approach was employed: (a) GeominQ rotation for the ESEM model and (b) target rotation, specifically targetT, for the B-ESEM model. This makes it possible to specify *a priori* the indicators of each factor and the free estimation of cross-loadings (Asparouhov and Muthén, 2009). Both models were based on the three-factor related model by Jonsén et al. (2010), as it was the model with the best fit and theoretical support with respect to the other CFA models. In addition, following the recommendation of Marsh et al. (2014) the ESEM and B-ESEM models were compared with the CFA models.

**FIGURE 1:**
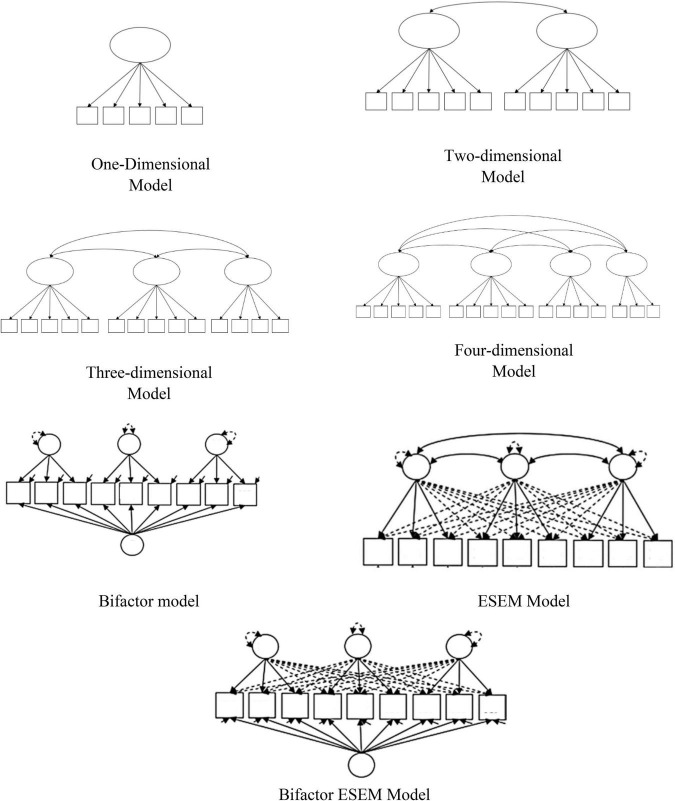
PIL models evaluated.

To evaluate the fit of the models, we followed the typical interpretation guidelines: the chi-squared test (χ^2^), the RMSEA index and the SRMR index, where values below 0.05 indicate a good fit of the model; whereas values between 0.05 and 0.08 express an acceptable fit (Kline, 2015). Likewise, the CFI and TLI indices were used, where values above 0.90 and 0.95 indicate an acceptable and good fit, respectively (Schumacker and Lomax, 2015). The difference between the 23 estimated models was not tested as the models employed different numbers of items and factors (from one factor to four factors). This makes the results of the chi-square difference test uninterpretable. Even in terms of information criterion indices such as AIC, it might be difficult to obtain a value from a comparison, as models with more items would have a higher power of rejection than models with fewer items. To evaluate the internal consistency of the scale, the omega coefficient was used, where a value greater than 0.70 was considered acceptable (McDonald, 1999, Viladrich et al., 2017). Figure 1 shows the graphical representations of the models tested (one-dimensional, two-dimensional, three-dimensional, four-dimensional, bifactor model, ESEM models and ESEM-Bifactor model).

Measurement invariance of PIL by participant nationality (country) was assessed through two methodological approaches: (a) exact measurement invariance (traditional approach) and (b) approximate measurement invariance (AMI). The traditional approach considers that the factorial weights and intercepts between groups are zero, so that the parameters must be equal to show invariance between groups. On the other hand, for the AMI approach, the factorial weights and intercepts are not necessarily equal between groups that have different cultural characteristics, allowing small differences in the parameters (Byrne and van de Vijver, 2017; Lomazzi, 2018; Fischer and Karl, 2019).

For the evaluation of invariance based on the traditional approach, we used Multi-Group Confirmatory Factor Analysis (MGCFA), which proposes a set of hierarchical variance models: configural invariance (reference model), metric invariance (where factor loadings are equal), scalar invariance (where factor loadings and intercepts are equal) and strict invariance (where factor loadings, intercepts and residuals are equal). The comparison of the sequence of models was carried out based on two strategies: first, a formal statistical test, based on the chi-square difference (Δχ^2^), where non-significant values (*p* > 0.05) indicate invariance between groups. Second, we used the differences in the CFI index (ΔCFI), where values less than <0.010 suggest model invariance between groups (Chen, 2007). We also used the RMSEA differences (ΔRMSEA), where values less than <0.015 indicate model invariance (Chen, 2007).

On the other hand, the evaluation of the invariance based on the AMI method used the Multi-Group Factor Analysis Alignment (Asparouhov and Muthén, 2014). Here, an unrestricted configural model was initially fitted to each of the groups. This configural model was then optimized with a component loss function to minimize the invariance in factor means and factor variances in each group (Asparouhov and Muthén, 2014). The invariance tolerance criteria were established as follows, for the factorial weights (λ = 0.40) and the intercepts (ν = 0.20) (Robitzsch, 2020). Likewise, the power of alignment was set at 0.25 for both parameters (Fischer and Karl, 2019). Parameter invariance was evaluated based on the R2 index, which is the average of the specific *R*^2^ of each parameter, where values close to 1 indicate a high degree of invariance, while values close to 0 indicate lower invariance (Asparouhov and Muthén, 2014). To consider a scale as non-invariant, a limit of 25% was established to evaluate the percentage of non-invariant parameters (λ and ν) (Asparouhov and Muthén, 2014).

All statistical analyses were carried out using the “lavaan” package (Rosseel, 2012) for the CFA, the “semTools” package (Jorgensen et al., 2018) for the factorial invariance, the “sirt” package (Robitzsch, 2020) for the Alignment method. In all cases, the RStudio environment was used (RStudio Team, 2018). The R codes and data are available in the open access repository OSF: https://osf.io/pd8n4/.

## Results

### Validity based on the internal structure and reliability of the Purpose in Life Test

The item 13 (“I am: Not very responsible—Very responsible”) has the highest average score in all countries and item 7 (“After retirement, I would like to: Do some things that have interested me—Laze around for the rest of my life”) has the lowest average score in most countries. Regarding the skewness and kurtosis indices, it can be seen that the items present adequate indices in most countries (As < ± 2; Ku < ± 7) according to the criteria of Finney and DiStefano (2013). This can be seen in the Supplementary material 1.

Table 2 shows the different models reported in the scientific literature for the PIL scale. As can be seen, the original model and its variants (1a–1d) do not show adequate adjustment indices in all countries. Similarly, a model of two related factors in its various variants (2a–2d) also does not show adequate fit indices across countries. With respect to a model of three related factors, only models 3c and 3f show acceptable fit indices in all countries. However, model 3f presents estimation problems (“covariance matrix of latent variables is not positive definite”) in the countries of Argentina, Colombia, El Salvador, and Uruguay. It can also be seen that a model with four related factors does not show adequate adjustment indexes in the countries. Table 3 shows that the items of model 3c have a high factorial weight in their corresponding dimension (>0.70) in most countries. It can also be seen that there is a significant relationship between the dimensions of the scale in all the countries. Therefore, model 3c was used as the basis for estimating the Bi-factor, ESEM and B-ESEM models.

**TABLE 2:** Fit indices, factorial weights and reliability of the models by country of participants.

Factor	Modelo	Estudio	Modelos/Ítems	País	χ^2^	df	*p*	CFI	TLI	SRMR	RMSEA [90% CI]
1 factor	1a	Crumbaugh and Maholick (1964) Brunelli et al. (2012) Simkin et al. (2018)	Meaning in life: 1–20	Argentina	2904.41	170	<0.001	0.92	0.92	0.061	0.109 [0.105–0.112]
				Colombia	839.88	170	<0.001	0.94	0.94	0.074	0.112 [0.104–0.119]
				Ecuador	2476.41	170	<0.001	0.88	0.87	0.097	0.133 [0.128–0.137]
				El Salvador	1138.88	170	<0.001	0.88	0.86	0.107	0.136 [0.129–0.144]
				Mexico	2146.85	170	<0.001	0.92	0.91	0.072	0.113 [0.109–0.118]
				Paraguay	600.51	170	<0.001	0.94	0.94	0.080	0.102 [0.093–0.111]
				Uruguay	874.74	170	<0.001	0.94	0.93	0.062	0.102 [0.095–0.109]
	1b	Marsh et al. (2003)	Meaning in life: 1–6, 8–13, 16–20	Argentina	2674.85	119	<0.001	0.92	0.91	0.061	0.126 [0.122–0.130]
				Colombia	748.44	119	<0.001	0.94	0.93	0.068	0.129 [0.121–0.138]
				Ecuador	2133.84	119	<0.001	0.89	0.87	0.085	0.148 [0.143–0.154]
				El Salvador	861.88	119	<0.001	0.89	0.88	0.088	0.142 [0.134–0.151]
				Mexico	1760.45	119	<0.001	0.93	0.92	0.062	0.124 [0.119–0.129]
				Paraguay	521.82	119	<0.001	0.95	0.94	0.076	0.118 [0.108–0.128]
				Uruguay	789.50	119	<0.001	0.94	0.93	0.060	0.119 [0.111–0.127]
	1c	Steger (2006)	Meaning in life: 1–6, 8–13, 16–17, 19–20	Argentina	2617.39	104	<0.001	0.92	0.91	0.063	0.133 [0.129–0.138]
				Colombia	711.31	104	<0.001	0.94	0.93	0.069	0.136 [0.127–0.145]
				Ecuador	1755.57	104	<0.001	0.90	0.89	0.077	0.144 [0.138–0.149]
				El Salvador	630.76	104	<0.001	0.92	0.91	0.077	0.128 [0.119–0.138]
				Mexico	1540.21	104	<0.001	0.93	0.92	0.059	0.124 [0.118–0.129]
				Paraguay	466.12	104	<0.001	0.95	0.94	0.074	0.120 [0.109–0.131]
				Uruguay	706.54	104	<0.001	0.94	0.93	0.060	0.121 [0.112–0.129]
	1d	Tibaldi Nascimento and Lebre Dias (2019)	Meaning in life: 1–6, 8–10, 12, 16, 19, 20	Argentina	2059.72	65	<0.001	0.92	0.90	0.066	0.150 [0.145–0.156]
				Colombia	518.06	65	<0.001	0.95	0.94	0.070	0.149 [0.137–0.161]
				Ecuador	1153.37	65	<0.001	0.92	0.91	0.074	0.147 [0.140–0.155]
				El Salvador	389.69	65	<0.001	0.94	0.93	0.072	0.127 [0.115–0.140]
				Mexico	1007.61	65	<0.001	0.94	0.93	0.060	0.127 [0.120–0.134]
				Paraguay	267.37	65	<0.001	0.96	0.95	0.062	0.113 [0.099–0.127]
				Uruguay	547.26	65	<0.001	0.94	0.93	0.062	0.136 [0.126–0.147]
2 factores	2a	Walters and Klein (1980)	Despair: 1, 3–4, 6, 8–9, 11–12, 20 Enthusiasm: 2, 5, 17–19	Argentina	1899.76	76	<0.001	0.94	0.92	0.053	0.133 [0.128–0.138]
				Colombia	394.48	76	<0.001	0.97	0.96	0.050	0.115 [0.104–0.127]
				Ecuador	624.02	76	<0.001	0.97	0.96	0.045	0.097 [0.090–0.104]
				El Salvador	296.93	76	<0.001	0.97	0.96	0.055	0.097 [0.086–0.109]
				Mexico	688.57	76	<0.001	0.97	0.96	0.040	0.094 [0.088–0.101]
				Paraguay	238.75	76	<0.001	0.98	0.97	0.048	0.094 [0.081–0.107]
				Uruguay	582.10	76	<0.001	0.95	0.94	0.058	0.129 [0.120–0.139]
	2b	Dufton and Perlman (1986)	Life satisfaction: 1–2, 5–6, 9–10, 19 Purpose in life: 3–4, 8, 11–12, 17, 20	Argentina	2104.75	76	<0.001	0.93	0.92	0.059	0.140 [0.135–0.145]
				Colombia	520.19	76	<0.001	0.96	0.95	0.062	0.136 [0.125–0.147]
				Ecuador	1576.74	76	<0.001	0.91	0.89	0.080	0.160 [0.153–0.167]
				El Salvador	582.85	76	<0.001	0.92	0.90	0.078	0.147 [0.136–0.158]
				Mexico	1373.11	76	<0.001	0.93	0.92	0.060	0.137 [0.131–0.144]
				Paraguay	391.95	76	<0.001	0.95	0.94	0.070	0.131 [0.118–0.144]
				Uruguay	545.77	76	<0.001	0.95	0.94	0.055	0.124 [0.115–0.134]
	2c	Molcar and Stuempfig (1988)	General meaning in life: 3–4, 7–9, 11, 13, 17, 20 Exciting daily life: 1–2, 5, 10, 12, 14, 18–19	Argentina	2122.19	118	<0.001	0.94	0.93	0.056	0.112 [0.108–0.116]
				Colombia	558.61	118	<0.001	0.95	0.95	0.063	0.109 [0.100–0.118]
				Ecuador	2100.23	118	<0.001	0.88	0.86	0.097	0.148 [0.142–0.153]
				El Salvador	873.95	118	<0.001	0.89	0.87	0.100	0.144 [0.135–0.153]
				Mexico	1738.44	118	<0.001	0.92	0.91	0.069	0.123 [0.118–0.128]
				Paraguay	510.59	118	<0.001	0.94	0.93	0.081	0.117 [0.107–0.128]
				Uruguay	682.23	118	<0.001	0.95	0.94	0.059	0.109 [0.102–0.118]
	2d	Shek (1988)	Existence: 1, 2, 3, 4, 5, 6, 8, 9, 11, 12, 13, 16, 17, 18, 19, 20 Death: 7, 10, 14, 15	Argentina	2910.46	169	<0.001	0.92	0.92	0.060	0.109 [0.106–0.113]
				Colombia	836.71	169	<0.001	0.94	0.93	0.073	0.112 [0.104–0.119]
				Ecuador	2443.53	169	<0.001	0.88	0.87	0.093	0.132 [0.128–0.137]
				El Salvador	1135.51	169	<0.001	0.88	0.86	0.105	0.136 [0.129–0.144]
				Mexico	2133.61	169	<0.001	0.92	0.91	0.071	0.113 [0.109–0.118]
				Paraguay	603.65	169	<0.001	0.94	0.94	0.079	0.103 [0.094–0.112]
				Uruguay	877.81	169	<0.001	0.94	0.93	0.062	0.103 [0.096–0.109]
	2e	McGregor and Little (1998)	Happiness: 1–2, 5, 8–9, 19 Meaning: 3, 17, 20	Argentina	1038.07	26	<0.001	0.94	0.91	0.068	0.169 [0.161–0.178]
				Colombia	232.54	26	<0.001	0.96	0.94	0.067	0.159 [0.140–0.178]
				Ecuador	862.27	26	<0.001	0.89	0.86	0.092	0.204 [0.193–0.216]
				El Salvador	300.91	26	<0.001	0.91	0.87	0.094	0.185 [0.167–0.204]
				Mexico	678.59	26	<0.001	0.94	0.91	0.065	0.167 [0.156–0.178]
				Paraguay	195.92	26	<0.001	0.94	0.92	0.077	0.164 [0.143–0.186]
				Uruguay	256.39	26	<0.001	0.96	0.95	0.055	0.149 [0.133–0.166]
	2f	Waisberg and Starr (1999)	Life with meaning: 3–4, 6, 8–13, 16–17, 20 Interest of the everyday: 1–2, 5, 9, 18–19	Argentina	1863.54	117	<0.001	0.95	0.94	0.051	0.105 [0.101–0.109]
				Colombia	564.41	117	<0.001	0.96	0.95	0.057	0.110 [0.101–0.119]
				Ecuador	1983.58	117	<0.001	0.89	0.88	0.079	0.144 [0.138–0.149]
				El Salvador	733.89	117	<0.001	0.92	0.90	0.080	0.131 [0.122–0.140]
				Mexico	1531.65	117	<0.001	0.94	0.93	0.057	0.116 [0.111–0.121]
				Paraguay	511.40	117	<0.001	0.95	0.94	0.073	0.118 [0.107–0.128]
				Uruguay	658.39	117	<0.001	0.95	0.94	0.056	0.108 [0.100–0.116]
	2g	Morgan and Farsides (2009)	Exciting life: 2, 5, 7, 10, 17, 18–19 Purposeful life: 3, 8, 20	Argentina	943.59	34	<0.001	0.94	0.92	0.054	0.140 [0.133–0.148]
				Colombia	149.28	34	<0.001	0.98	0.97	0.047	0.104 [0.087–0.121]
				Ecuador	306.57	34	<0.001	0.97	0.96	0.045	0.102 [0.092–0.113]
				El Salvador	177.57	34	<0.001	0.96	0.94	0.062	0.117 [0.100–0.134]
				Mexico	356.18	34	<0.001	0.97	0.96	0.045	0.102 [0.093–0.112]
				Paraguay	117.58	34	<0.001	0.97	0.96	0.053	0.101 [0.081–0.121]
				Uruguay	308.45	34	<0.001	0.95	0.94	0.062	0.142 [0.128–0.157]
	2h	García-Alandete et al. (2013)	Satisfaction and meaning in life: 1–2, 5–6, 9, 11 Goals and purposes in life: 3, 7, 17, 20	Argentina	1176.11	34	<0.001	0.93	0.91	0.066	0.157 [0.150–0.165]
				Colombia	285.05	34	<0.001	0.95	0.94	0.069	0.153 [0.137–0.170]
				Ecuador	743.38	34	<0.001	0.92	0.89	0.082	0.165 [0.154–0.175]
				El Salvador	328.88	34	<0.001	0.91	0.88	0.097	0.168 [0.152–0.185]
				Mexico	665.56	34	<0.001	0.94	0.92	0.066	0.143 [0.134–0.153]
				Paraguay	220.68	34	<0.001	0.94	0.93	0.079	0.150 [0.132–0.170]
				Uruguay	315.55	34	<0.001	0.96	0.94	0.064	0.144 [0.130–0.159]
	2i	Hayashi and Esmerelles (2017)	Emoción en la vida: 2,5,61,9,19 Responsabilidad individual: 13, 18, 20, 11	Argentina	1169.17	34	<0.001	0.93	0.90	0.067	0.157 [0.149–0.165]
				Colombia	312.60	34	<0.001	0.95	0.93	0.070	0.161 [0.145–0.178]
				Ecuador	910.56	34	<0.001	0.89	0.86	0.091	0.183 [0.173–0.193]
				El Salvador	392.40	34	<0.001	0.89	0.86	0.095	0.185 [0.169–0.202]
				Mexico	793.07	34	<0.001	0.93	0.91	0.064	0.157 [0.148–0.167]
				Paraguay	195.79	34	<0.001	0.95	0.93	0.074	0.140 [0.121–0.159]
				Uruguay	364.93	34	<0.001	0.94	0.92	0.066	0.156 [0.142–0.171]
3 factores	3a	Magaña Valladares et al. (2004)	Perception of meaning in life: 1–5, 7–10, 13, 17, 19–20 Life satisfaction: 6, 11–12, 16, 18 Freedom and control of life: 14–15	Argentina	2671.91	167	<0.001	0.93	0.92	0.058	0.105 [0.102–0.109]
				Colombia	790.96	167	<0.001	0.95	0.94	0.072	0.109 [0.101–0.116]
				Ecuador	2460.74	167	<0.001	0.88	0.87	0.096	0.133 [0.129–0.138]
				El Salvador	1124.22	167	<0.001	0.88	0.86	0.106	0.136 [0.129–0.144]
				Mexico	2062.27	167	<0.001	0.92	0.91	0.071	0.112 [0.108–0.116]
				Paraguay	589.00	167	<0.001	0.95	0.94	0.079	0.102 [0.093–0.111]
				Uruguay	807.53	167	<0.001	0.95	0.94	0.059	0.098 [0.091–0.105]
	3b	Risco (2009)	Value of life: 1, 4, 6, 9, 10–12 Capacity of meaning: 2, 5, 7, 14–15, 17–19 Goals and responsibility: 3, 8, 13, 20	Argentina	2127.93	149	<0.001	0.94	0.94	0.051	0.099 [0.095–0.103]
				Colombia	566.30	149	<0.001	0.96	0.96	0.059	0.094 [0.086–0.102]
				Ecuador	1476.94	149	<0.001	0.93	0.92	0.069	0.108 [0.103–0.113]
				El Salvador	628.77	149	<0.001	0.94	0.93	0.076	0.102 [0.094–0.111]
				Mexico	1400.24	149	<0.001	0.95	0.94	0.057	0.096 [0.092–0.101]
				Paraguay	469.12	149	<0.001	0.96	0.95	0.067	0.094 [0.085–0.104]
				Uruguay	697.78	149	<0.001	0.95	0.95	0.056	0.096 [0.089–0.103]
	3c	Jonsén et al. (2010)	Meaning of existence: 1, 3–4, 6, 8–9, 11, 20 Freedom to make meaning in daily life: 10, 14–15, 17–19 Find meaning before future challenges: 2, 5, 7	Argentina	1611.73	116	<0.001	0.95	0.95	0.048	0.097 [0.093–0.102]
				Colombia	441.28	116	<0.001	0.97	0.96	0.056	0.094 [0.085–0.104]
				Ecuador	798.48	116	<0.001	0.96	0.95	0.055	0.087 [0.082–0.092]
				El Salvador	461.37	116	<0.001	0.95	0.94	0.071	0.098 [0.089–0.108]
				Mexico	792.70	116	<0.001	0.97	0.96	0.048	0.080 [0.075–0.086]
				Paraguay	305.14	116	<0.001	0.97	0.97	0.057	0.082 [0.071–0.093]
				Uruguay	478.19	116	<0.001	0.97	0.96	0.050	0.088 [0.080–0.097]
	3d	Martínez Ortiz et al. (2012)	Establecer metas: 7, 10, 11, 13, 16, 17, 18, 19, 20 Componente hedónico de la vida: 1, 2, 3, 4, 5, 6, 9 Sensación de logro: 8, 12, 14, 15	Argentina	2851.77	167	<0.001	0.93	0.92	0.060	0.109 [0.105–0.112]
				Colombia	836.70	167	<0.001	0.94	0.93	0.074	0.113 [0.105–0.120]
				Ecuador	2402.47	167	<0.001	0.89	0.87	0.096	0.132 [0.127–0.136]
				El Salvador	1094.02	167	<0.001	0.88	0.87	0.106	0.134 [0.127–0.142]
				Mexico	2071.21	167	<0.001	0.92	0.91	0.071	0.112 [0.108–0.117]
				Paraguay	582.50	167	<0.001	0.95	0.94	0.079	0.101 [0.092–0.110]
				Uruguay	859.43	167	<0.001	0.94	0.93	0.061	0.102 [0.095–0.109]
	3e	Gottfried (2016)	Factor 1: 4, 7, 8, 9, 11, 12, 13, 14, 17, 18, 20 Factor 2: 1, 2, 3, 5, 15, 19 Factor 3: 6, 10, 15, 16	Argentina	2353.49	166	<0.001	0.94	0.93	0.055	0.098 [0.095–0.102]
				Colombia	756.74	166	<0.001	0.95	0.94	0.070	0.106 [0.099–0.114]
				Ecuador	2433.59	166	<0.001	0.88	0.87	0.096	0.133 [0.128–0.138]
				El Salvador	1108.27	166	<0.001	0.88	0.86	0.105	0.136 [0.128–0.143]
				Mexico	2032.42	166	<0.001	0.92	0.92	0.070	0.112 [0.107–0.116]
				Paraguay	574.11	166	<0.001	0.95	0.94	0.077	0.101 [0.092–0.110]
				Uruguay	735.66	166	<0.001	0.95	0.95	0.056	0.093 [0.086–0.100]
	3f	Armas et al. (2018)	Valor de Vida: 20, 9, 1, 4, 8, 3, 11, 12, 6 Capacidad de significado: 2, 19, 5, 18, 17, 10, 14, 7 Responsabilidad y sentido de vida: 13, 16	Argentina^a^	2526.22	149	<0.001	0.93	0.92	0.053	0.108 [0.105–0.112]
				Colombia^a^	534.52	149	0.000	0.97	0.96	0.053	0.090 [0.082–0.099]
				Ecuador	992.83	149	<0.001	0.97	0.95	0.052	0.086 [0.081–0.091]
				El Salvador^a^	508.19	149	<0.001	0.95	0.95	0.065	0.088 [0.080–0.097]
				Mexico	1043.15	149	<0.001	0.96	0.96	0.045	0.082 [0.077–0.086]
				Paraguay	395.97	149	<0.001	0.97	0.96	0.059	0.083 [0.073–0.093]
				Uruguay^a^	770.16	149	<0.001	0.95	0.94	0.057	0.102 [0.095–0.109]
4 factores	4a	Huamani and Arias (2018)	Percepción de sentido: 6, 9, 10, 11, 12, 16, 17 Experiencia de sentido: 1, 2, 5, 20 Metas y tareas: 3, 7, 8, 13 Dialéctica/destino y libertad: 14, 15, 18	Argentina	2005.33	129	<0.001	0.93	0.92	0.056	0.103 [0.099–0.107]
				Colombia	641.53	129	<0.001	0.94	0.93	0.069	0.112 [0.104–0.121]
				Ecuador	1983.45	129	<0.001	0.88	0.86	0.094	0.137 [0.131–0.142]
				El Salvador	824.18	129	<0.001	0.89	0.87	0.100	0.132 [0.124–0.141]
				Mexico	1503.67	129	<0.001	0.93	0.91	0.067	0.109 [0.104–0.114]
				Paraguay	470.96	129	<0.001	0.94	0.93	0.077	0.104 [0.094–0.115]
				Uruguay	666.04	129	<0.001	0.95	0.94	0.060	0.102 [0.095–0.110]
Bi-factor model	5^b^	Bi-factor model with three specific factors	Meaning of existence: 1, 3–4, 6, 8–9, 11, 20 Freedom to make meaning in daily life: 10, 14–15, 17–19 Find meaning before future challenges: 2, 5, 7	Argentina^a^	-	-	-	-	-	-	-
				Colombia^a^	-	-	-	-	-	-	-
				Ecuador^a^	-	-	-	-	-	-	-
				El Salvador^a^	-	-	-	-	-	-	-
				Mexico	676.37	102	<0.001	0.97	0.96	0.042	0.079 [0.073–0.085]
				Paraguay^a^	-	-	-	-	-	-	-
				Uruguay^a^	-	-	-	-	-	-	-
ESEM model	6^b^	Three factor ESEM model	Meaning of existence: 1, 3–4, 6, 8–9, 11, 20 Freedom to make meaning in daily life: 10, 14–15, 17–19 Find meaning before future challenges: 2, 5, 7	Argentina	861.73	88	<0.001	0.98	0.96	0.031	0.080 [0.076–0.085]
				Colombia	254.58	88	<0.001	0.98	0.97	0.032	0.077 [0.066–0.089]
				Ecuador	323.93	88	<0.001	0.98	0.98	0.025	0.059 [0.052–0.066]
				El Salvador	205.92	88	<0.001	0.98	0.97	0.034	0.066 [0.054–0.078]
				Mexico	457.21	88	<0.001	0.98	0.97	0.027	0.068 [0.062–0.072]
				Paraguay	207.09	88	<0.001	0.98	0.97	0.040	0.075 [0.062–0.088]
				Uruguay	321.71	88	<0.001	0.98	0.97	0.039	0.082 [0.072–0.091]
Bifactor ESEM Model	7^b^	Bifactor ESEM model with three specific factors	Meaning of existence: 1, 3–4, 6, 8–9, 11, 20 Freedom to make meaning in daily life: 10, 14–15, 17–19 Find meaning before future challenges: 2, 5, 7	Argentina	476.17	74	<0.001	0.98	0.96	0.016	0.036 [0.033–0.039]
				Colombia	104.49	74	<0.001	0.98	0.96	0.023	0.036 [0.018–0.051]
				Ecuador	106.06	74	<0.001	0.99	0.98	0.018	0.024 [0.012–0.033]
				El Salvador	86.21	74	<0.001	0.99	0.98	0.026	0.023 [0.000–0.042]
				Mexico	150.24	74	<0.001	0.98	0.96	0.019	0.034 [0.026–0.042]
				Paraguay	97.41	74	<0.001	0.98	0.96	0.029	0.036 [0.010–0.054]
				Uruguay	140.85	74	<0.001	0.93	0.88	0.027	0.048 [0.035–0.059]

χ^2^ = Chi square; df, degrees of freedom; SRMR, Standardized Root Mean Square Residual; TLI, Tucker-Lewis Index; CFI, Comparative Fit Index; RMSEA, Root Mean Square Error of Approximation; ^a^covariance matrix of latent variables is not positive definite; ^b^based on the model by Jonsén et al. (2010).

**TABLE 3:** Factorial weight and reliability of the PIL scale by country.

Model	Country	Factors	1	3	4	6	8	9	11	20	10	14	15	17	18	19	2	5	7	F1	F2	F3	ω
Model 3c	Argentina	F1	0.70	0.70	0.87	0.74	0.73	0.86	0.79	0.87	-	-	-	-	-	-	-	-	-	-	−0.89	−0.71	0.93
		F2	-	-	-	-	-	-	-	-	0.81	0.42	0.11	0.81	0.63	0.79	-	-	-		−	0.78	0.69
		F3	-	-	-	-	-	-	-	-	-	-	-	-	-	-	0.84	0.83	0.31			−	0.72
	Colombia	F1	0.74	0.80	0.90	0.81	0.75	0.89	0.82	0.83	-	-	-	-	-	-	-	-	-	−	−0.84	−0.75	0.94
		F2	-	-	-	-	-	-	-	-	0.78	0.60	0.16	0.82	0.78	0.82	-	-	-		−	0.90	0.76
		F3	-	-	-	-	-	-	-	-	-	-	-	-	-	-	0.85	0.82	0.37			−	0.74
	Ecuador	F1	0.60	0.76	0.87	0.79	0.73	0.88	0.75	0.83	-	-	-	-	-	-	-	-	-	−	−0.67	−0.56	0.91
		F2	-	-	-	-	-	-	-	-	0.82	0.55	0.14	0.84	0.73	0.78	-	-	-		−	0.84	0.75
		F3	-	-	-	-	-	-	-	-	-	-	-	-	-	-	0.76	0.68	0.55			−	0.70
	El Salvador	F1	0.61	0.79	0.90	0.80	0.75	0.90	0.81	0.87	-	-	-	-	-	-	-	-	-	−	−0.69	−0.58	0.92
		F2	-	-	-	-	-	-	-	-	0.84	0.59	0.23	0.80	0.80	0.84	-	-	-		−	0.81	0.77
		F3	-	-	-	-	-	-	-	-	-	-	-	-	-	-	0.67	0.80	0.53			−	0.71
	Mexico	F1	0.67	0.72	0.90	0.79	0.75	0.88	0.83	0.86	-	-	-	-	-	-	-	-	-	−	−0.78	−0.70	0.92
		F2	-	-	-	-	-	-	-	-	0.80	0.56	0.21	0.85	0.71	0.86	-	-	-		−	0.82	0.75
		F3	-	-	-	-	-	-	-	-	-	-	-	-	-	-	0.81	0.81	0.39			−	0.67
	Paraguay	F1	0.66	0.68	0.89	0.81	0.72	0.86	0.87	0.88	-	-	-	-	-	-	-	-	-	−	−0.79	−0.68	0.92
		F2	-	-	-	-	-	-	-	-	0.87	0.46	0.21	0.86	0.73	0.83	-	-	-		−	0.81	0.75
		F3	-	-	-	-	-	-	-	-	-	-	-	-	-	-	0.83	0.73	0.10			−	0.52
	Uruguay	F1	0.80	0.74	0.86	0.72	0.72	0.89	0.83	0.90	-	-	-	-	-	-	-	-	-	−	−0.92	−0.76	0.93
		F2	-	-	-	-	-	-	-	-	0.82	0.51	0.11	0.85	0.68	0.78	-	-	-		−	0.76	0.73
		F3	-	-	-	-	-	-	-	-	-	-	-	-	-	-	0.88	0.83	0.41			−	0.74
ESEM Model	Argentina	F1	0.37	0.83	0.72	0.53	0.54	0.62	0.54	0.86	−0.23	0.20	0.28	−0.32	0.05	−0.18	0.06	0.05	−0.01	-	−0.42	−0.71	-
		F2	−0.51	−0.14	−0.12	−0.13	−0.17	−0.16	−0.07	−0.16	0.17	0.03	0.17	0.18	0.11	0.40	0.79	0.74	0.06		-	0.36	-
		F3	0.01	0.22	−0.14	−0.18	−0.13	−0.20	−0.28	0.05	0.53	0.68	0.28	0.42	0.70	0.37	0.21	0.23	0.25			-	-
	Colombia	F1	0.40	0.62	0.68	0.71	0.55	0.68	0.75	0.67	−0.14	0.09	0.31	−0.13	−0.08	−0.08	−0.09	0.03	0.19	-	−0.42	−0.61	-
		F2	−0.63	0.10	−0.09	−0.29	−0.21	−0.28	−0.18	0.01	0.31	0.27	0.18	0.08	0.18	0.53	0.67	0.67	−0.04		-	0.49	-
		F3	0.14	−0.37	−0.25	0.06	−0.12	−0.07	−0.01	−0.26	0.46	0.52	0.31	0.72	0.64	0.36	0.19	0.28	0.61			-	-
	Ecuador	F1	0.45	0.75	0.75	0.65	0.70	0.71	0.60	0.75	−0.18	0.10	0.18	−0.20	−0.08	−0.09	0.08	0.17	0.05	-	−0.31	−0.49	-
		F2	0.18	−0.29	−0.15	0.02	−0.08	−0.06	0.02	−0.25	0.48	0.55	0.32	0.70	0.71	0.54	0.09	0.19	0.59		-	0.49	-
		F3	−0.38	0.17	−0.11	−0.26	−0.03	−0.25	−0.27	0.04	0.30	0.19	0.02	0.09	0.07	0.29	0.77	0.67	0.04			-	-
	El Salvador	F1	0.37	0.79	0.76	0.73	0.73	0.74	0.67	0.79	−0.33	0.13	0.35	−0.18	0.02	−0.11	0.23	0.01	0.12	-	−0.39	−0.52	-
		F2	0.15	−0.15	−0.19	0.09	−0.02	−0.06	−0.09	−0.21	0.49	0.58	0.34	0.72	0.76	0.58	0.06	0.23	0.63		-	0.43	-
		F3	−0.48	0.09	−0.07	−0.23	−0.06	−0.21	−0.15	0.03	0.15	0.24	0.29	0.05	0.22	0.30	0.90	0.61	0.04			-	-
	Mexico	F1	0.51	0.65	0.76	0.74	0.70	0.70	0.72	0.80	−0.15	0.06	0.31	−0.21	−0.04	−0.13	0.01	0.08	0.20	-	−0.50	−0.64	-
		F2	0.08	−0.17	−0.06	−0.18	−0.19	−0.13	−0.25	−0.35	0.48	0.75	0.29	0.66	0.76	0.58	0.04	0.17	0.26		-	0.63	-
		F3	−0.31	0.03	−0.17	0.06	0.07	−0.14	0.07	0.21	0.25	−0.08	0.26	0.07	−0.02	0.24	0.79	0.73	0.34			-	-
	Paraguay	F1	0.25	0.35	0.62	0.69	0.46	0.57	0.72	0.58	−0.35	−0.04	0.22	−0.27	−0.27	−0.19	0.18	0.06	−0.10	-	−0.38	0.48	-
		F2	0.14	−0.09	−0.12	−0.15	0.15	−0.08	−0.14	−0.18	0.59	0.45	0.31	0.75	0.65	0.56	0.48	0.43	0.37		-	−0.47	-
		F3	0.69	0.38	0.32	0.13	0.52	0.38	0.16	0.29	−0.08	−0.05	−0.16	−0.01	0.06	−0.27	−0.57	−0.39	0.38			-	-
	Uruguay	F1	0.30	−18	0.37	0.52	0.36	0.57	0.58	0.40	−0.54	−0.20	0.05	−0.38	−0.49	−0.34	−0.03	0.03	0.11	-	−0.33	0.55	-
		F2	−0.41	0.27	0.05	−0.18	−0.07	−0.18	−0.04	0.00	0.35	0.15	0.28	0.26	0.20	0.41	0.72	0.74	0.25		-	−0.65	-
		F3	0.27	1.19	0.60	0.15	0.42	0.27	0.32	0.60	−0.06	−0.19	0.12	−0.28	−0.09	−0.14	−0.13	−0.15	−0.20			-	-
Bi-ESEM Mode	Argentina	F1	−0.04	−0.56	−0.08	−16	−0.11	0.00	0.15	−0.29	−0.13	−0.05	0.03	0.09	0.04	0.05	−0.03	−0.01	0.07	-	-	-	-
		F2	0.06	0.02	0.07	0.07	−0.03	0.03	−0.01	−0.03	0.21	0.39	0.19	0.27	0.53	0.22	0.08	0.11	0.20	-	-	-	-
		F3	−0.29	0.05	0.12	0.07	0.02	0.06	0.10	0.09	0.02	0.01	0.17	0.01	0.03	0.24	0.60	0.56	0.04	-	-	-	-
		FG	0.65	0.63	0.84	0.75	0.69	0.82	0.76	0.82	−0.70	−0.31	−0.03	−0.69	−0.51	−0.67	−0.58	−0.56	−0.15	-	-	-	-
	Colombia	F1	−0.05	−0.61	−0.39	−0.11	−0.29	−0.28	−0.13	−0.52	−0.07	−0.01	−0.05	0.14	0.09	−0.00	−0.06	−0.08	0.02	-	-	-	-
		F2	−0.14	0.12	0.00	−0.14	0.01	−0.06	−0.17	0.06	−0.35	−0.32	−0.29	−0.42	−0.38	−0.26	−0.14	−0.18	−0.39	-	-	-	-
		F3	0.34	0.05	−0.13	−0.16	0.05	−0.03	−0.29	0.00	0.05	−0.11	−0.19	0.08	−0.01	−0.27	−0.37	−0.38	0.01	-	-	-	-
		FG	0.70	0.56	0.76	0.79	0.66	0.80	0.79	0.64	−0.69	−0.49	−0.09	−0.67	−0.66	−0.72	0.69	−0.67	−0.18	-	-	-	-
	Ecuador	F1	0.46	0.67	0.64	0.50	0.58	0.60	0.48	0.63	−0.07	0.11	0.17	−0.07	0.01	−0.04	−0.06	0.04	0.12	-	-	-	-
		F2	−0.14	0.34	0.01	−0.25	0.04	−0.12	−0.18	0.12	−0.14	−0.25	−0.21	−0.38	−0.39	−0.29	−0.02	−0.08	−0.30	-	-	-	-
		F3	0.25	0.02	−0.06	−0.09	0.02	−0.05	0.02	−0.02	0.05	−0.04	−0.03	0.08	0.02	−0.14	−0.52	−0.35	0.09	-	-	-	-
		FG	0.38	0.32	0.52	0.59	0.39	0.61	0.50	0.45	−0.74	−0.51	−0.15	−0.70	−0.61	−0.67	−0.60	−0.56	−0.43	-	-	-	-
	El Salvador	F1	−0.50	−0.51	−0.60	−0.65	−0.58	−0.68	−0.55	−0.60	0.19	−0.07	−0.19	−0.08	−0.04	0.10	0.03	0.13	−0.23	-	-	-	-
		F2	−0.26	0.06	0.05	0.07	−.07	0.04	0.04	0.12	0.08	0.43	0.23	−0.01	0.31	0.25	−0.02	0.20	−0.01	-	-	-	-
		F3	0.28	−0.08	−0.09	0.08	0.02	−0.00	−0.01	−0.04	0.04	−0.02	−0.12	0.06	0.02	−0.08	−0.89	−0.33	0.02	-	-	-	-
		FG	−0.27	−0.50	−0.58	−0.42	−0.41	−0.55	−0.47	−0.55	0.71	0.49	0.24	0.83	0.77	0.74	0.48	0.51	0.52	-	-	-	-
	Mexico	F1	0.05	0.75	0.19	0.03	0.18	−0.01	0.09	0.28	0.07	0.00	0.06	−0.09	0.04	−0.05	0.05	−0.00	−0.02	-	-	-	-
		F2	−0.13	0.04	−0.09	−0.06	−0.02	−0.08	−0.00	0.03	−0.37	−0.48	−0.27	−0.50	−0.52	−0.41	−0.12	−0.20	−0.29	-	-	-	-
		F3	0.18	0.06	−0.03	−0.14	−0.08	−0.07	−0.12	−0.13	−0.11	−0.03	−0.20	−0.04	−0.04	−0.21	−0.48	−0.52	−0.25	-	-	-	-
		FG	0.64	0.57	0.82	0.76	0.68	0.84	0.76	0.77	−0.63	−0.36	−0.09	−0.63	−0.51	−0.65	−0.60	−0.57	−0.19	-	-	-	-
	Paraguay	F1	−0.57	−0.23	−0.46	−0.42	−0.56	−0.53	−0.42	−0.45	0.09	−0.00	−0.02	−0.11	−0.06	0.17	0.05	−0.00	−0.35	-	-	-	-
		F2	0.09	−0.03	−0.16	−0.01	0.05	0.05	−0.07	0.09	−0.21	−0.32	−0.42	−0.16	−0.34	−0.37	−0.15	0.02	0.19	-	-	-	-
		F3	0.29	0.06	−0.04	−0.12	0.04	0.01	−0.09	−0.09	−0.05	−0.03	−0.14	0.01	0.09	−0.11	−0.53	−0.44	0.07	-	-	-	-
		FG	0.41	0.52	0.73	0.65	0.45	0.62	0.69	0.66	−0.73	−0.34	−0.11	−0.83	−0.68	−0.70	−0.60	−0.57	−0.25	-	-	-	-
	Uruguay	F1	0.10	0.29	0.45	0.31	0.30	0.44	0.42	0.35	−0.20	0.03	0.32	−0.14	−0.11	−0.02	0.13	0.21	−0.03	-	-	-	-
		F2	−0.03	0.01	0.06	0.02	−0.03	0.04	0.11	0.11	−0.20	−0.24	−0.03	−0.29	−0.51	−0.27	0.17	0.16	−0.15	-	-	-	-
		F3	−0.04	−0.13	−0.03	0.08	0.14	−0.02	0.07	0.07	0.11	−0.09	−0.16	0.10	−0.03	0.05	0.23	0.21	0.64	-	-	-	-
		FG	0.80	0.63	0.70	0.63	0.68	0.75	0.69	0.79	−0.69	−0.46	−0.17	−0.71	−0.58	−0.72	−0.76	−0.77	−0.24	-	-	-	-

F1, meaning of existence; F2, freedom to make meaning in daily life; F3, will to find meaning in the face of future challenges; ω, McDonald’s Omega.

As shown in Table 2, the Bi-factor model could only be estimated in Mexico, since in the other countries it presented estimation problems (“covariance matrix of latent variables is not positive definite”). With respect to the last two models, it can be seen that the ESEM model shows acceptable fit indices and the B-ESEM model shows excellent fit indices in all countries. Nevertheless, it is important to evaluate the factor loadings estimated in the models before choosing the best model. Regarding the ESEM model, it can be seen in Table 3 that the specific factor meaning of existence is well defined in most countries, with 1 to 4 items that evidenced a cross-loading above 0.25 on other factors. The specific factor Freedom to make meaning in daily life is poorly defined in Argentina (|λ| = 0.17 a.40; M| λ| = 0.18), Colombia (|λ| = 0.08 a.53; M| λ| = 0.26) and Uruguay (|λ| = 0.15 a.41; M| λ| = 0.28). In addition, it can be seen that there are 2 to 5 items with cross-loadings greater than 0.25 in other factors. The third factor, Will to find meaning before future challenges, is poorly defined in Argentina (|λ| = 0.21 a.25; M| λ| = 0.23), Colombia (|λ| = 0.19 a.61; M| λ| = 0.36), Ecuador (|λ| = 0.04 a.77; M| λ| = 0.49), Paraguay (|λ| = 0.38 a −0.57; M| λ| = −0.19), and Uruguay (|λ| = −0.13 a −0.20; M| λ| = −0.16). It also shows 3 to 9 items with cross-loadings greater than 0.25 on other factors.

With respect to the B-ESEM model, Table 3 shows that the general factor is well defined in most of the countries, except in Ecuador and El Salvador, where the weight of the items of factor 1 was higher than the general factor. It can also be seen that the first specific factor is not well defined in Argentina (|

λ| = 0.00 a −0.56; M| λ| = 0.17), Colombia (|λ| = −0.05 a −0.61; M| λ| = 0.30), Mexico (|λ| = −0.01 a.75; M| λ| = 0.20) and Uruguay (|λ| = 0.10 a.45; M| λ| = 0.33). Similarly, the second specific factor is not well defined in Argentina (|λ| = 0.19 a.53; M| λ| = 0.30), Ecuador (|λ| = −0.14 a −0.39; M| λ| = 0.28), El Salvador (|λ| = −0.01 a.31; M| λ| = 0.22) and Uruguay (|λ| = −0.02 a −0.51; M| λ| = 0.26). With respect to the third specific factor, item 7 has a very low factorial weight in most of the countries, therefore, it shows problems of representativeness since only two items are left from it. In view of the above, model 3c was chosen to perform the following statistical analyses.

For the estimation of the reliability of the scale (model 3c), the results of the CFA were used. As can be seen in Table 4, the dimensions of the Meaning of existence (ω ≥ 0.92), Freedom to make meaning in daily life (ω ≥ 0.69) and Will to find meaning before future challenges (ω ≥ 0.60) show acceptable reliability estimates in most of the countries, except in Paraguay, specifically for the third factor (ω = 0.52).

**TABLE 4:** Fit indices of the invariance models by country of the participants.

Unidimensional model	χ^2^	df	*p*	SRMR	TLI	CFI	RMSEA	Δχ^2^	Δ df	*p*	Δ CFI	Δ RMSEA
Total sample	4014.22	116	<0.001	0.045	0.95	0.96	0.088	–	–	–	–	–
**By country**												
Configural	2470.06	812	<0.001	0.046	0.87	0.90	0.058	–	–	–	–	–
Metric	1820.33	896	<0.001	0.055	0.94	0.94	0.041	106.18	84	0.051	0.04	−0.02
Scalar	2225.99	980	<0.001	0.060	0.92	0.92	0.045	294.02	84	<0.001	−0.02	0.00
Strict	2478.55	1082	<0.001	0.067	0.92	0.91	0.046	205.73	102	<0.001	−0.01	0.00

χ^2^, Chi square; df, degrees of freedom; SRMR, Standardized Root Mean Square Residual; TLI, Tucker-Lewis Index; CFI, Comparative Fit Index; RMSEA, Root Mean Square Error of Approximation; Δχ^2^, differences in Chi square; Δdf, differences in degrees of freedom; ΔRMSEA, Change in Root Mean Square Error of Approximation; ΔCFI, Change in Comparative Fix Index. ^a^The intercept of items 1 and 2 was released.

### Factor invariance by country

Table 4 shows that the factor structure of the PIL did not show evidence of strict invariance based on MGCFA for the different countries evaluated, based on the sequence of invariance models proposed: metric invariance (ΔCFI = 0.04; ΔRMSEA = −0.02), scalar invariance (ΔCFI = −0.02; ΔRMSEA = 0.00) and strict invariance (ΔCFI = −0.01; ΔRMSEA = 0.00).

On the other hand, the analysis with the Alignment method showed that the factor structure of the PIL is invariant for the factor loadings (*R*^2^ = 0.99) and the intercepts of the items (*R*^2^ = 0.99) (Table 5). It is also observed that all factor weights are invariant (0%) across countries; although there are 12 non-invariant parameters with respect to the intercepts. However, at the general level, the percentage of non-invariant parameters is low (10.1%). The findings suggest the presence of approximate invariance at the PIL metric and scalar levels.

**TABLE 5:** ML invariance alignment (IA) in all seven countries.

Parameters	Items	*Med*	*SD*	Min	Max	Country							*R* ^2^	%
Factorial weight	1	0.86	0.11	0.75	1.06	1	2	3	4	5	6	7	0.99	0.0%
	2	−1.05	0.11	−1.06	−0.79	1	2	3	4	5	6	7		
	3	0.81	0.08	0.73	0.95	1	2	3	4	5	6	7		
	4	1.07	0.10	0.95	1.27	1	2	3	4	5	6	7		
	5	−0.99	0.09	−1.01	−0.73	1	2	3	4	5	6	7		
	6	1.09	0.10	0.88	1.14	1	2	3	4	5	6	7		
	7	−0.43	0.14	−0.48	−0.10	1	2	3	4	5	6	7		
	8	0.87	0.06	0.80	0.99	1	2	3	4	5	6	7		
	9	1.01	0.09	0.91	1.15	1	2	3	4	5	6	7		
	10	−1.14	0.07	−1.22	−1.07	1	2	3	4	5	6	7		
	11	1.14	0.08	1.07	1.29	1	2	3	4	5	6	7		
	14	−0.75	0.09	−0.90	−0.64	1	2	3	4	5	6	7		
	15	−0.17	0.08	−0.28	−0.06	1	2	3	4	5	6	7		
	17	−1.02	0.05	−1.07	−0.95	1	2	3	4	5	6	7		
	18	−0.87	0.07	−1.01	−0.86	1	2	3	4	5	6	7		
	19	−1.03	0.08	−1.15	−0.89	1	2	3	4	5	6	7		
	20	0.95	0.06	0.87	1.04	1	2	3	4	5	6	7		
Intercept	1	4.64	0.05	4.62	4.75	1	2	3	4	5	6	7	0.99	10.1%
	2	3.92	0.16	3.69	4.16	1	(2)	3	(4)	5	6	7		
	3	5.90	0.19	5.59	6.16	1	2	3	(4)	5	6	(7)		
	4	5.92	0.09	5.79	5.99	1	2	3	4	5	6	7		
	5	3.99	0.09	3.86	4.19	1	2	3	4	5	6	7		
	6	5.47	0.11	5.38	5.63	1	2	3	4	5	6	7		
	7	2.41	0.37	2.22	3.09	1	2	3	4	(5)	(6)	7		
	8	5.37	0.05	5.33	5.48	1	2	3	4	5	6	7		
	9	5.83	0.02	5.78	5.84	1	2	3	4	5	6	7		
	10	2.64	0.13	2.47	2.87	1	2	3	(4)	5	6	7		
	11	5.42	0.09	5.36	5.55	1	2	3	4	5	6	7		
	14	3.32	0.33	3.16	3.96	(1)	(2)	3	4	5	6	(7)		
	15	3.81	0.15	3.62	4.06	(1)	2	3	4	5	6	7		
	17	2.53	0.08	2.44	2.67	1	2	3	4	5	6	7		
	18	2.78	0.10	2.74	2.98	1	2	3	4	5	6	7		
	19	3.11	0.09	2.95	3.19	1	2	3	4	5	6	7		
	20	5.76	0.12	5.61	5.99	1	2	3	(4)	5	6	7		

% = Percentage of item parameters without invariance. Parentheses indicate that the parameter is not invariant for that specific group (country).

## Discussion

In seeking to respond to the need for a measure of life purpose that can be used in different countries, the present study used a data set from seven Latin American countries (Argentina, Colombia, Ecuador, El Salvador, Mexico, Paraguay, and Uruguay) to evaluate the MI and other psychometric properties of the PIL in Spanish. Overall, the results indicate that the PIL can be used to compare life purpose across some Latin American countries. This is particularly valuable because of the growing need to understand the life purpose of populations in different contexts. Assessing the psychometric properties and cross-cultural utility of the PIL contributes to a better understanding of the theoretical underpinnings of life purpose and provides researchers with information to guide the choice of appropriate indicators to validly and reliably measure this construct.

The PIL has received numerous criticisms due to the inconsistency in the factorial structure (Jonsén et al., 2010), which have their origin in the fact that initially Crumbaugh and Maholick (1964) did not provide information on the method of formulation and selection of the items nor on the statistical treatment of the items (Davies et al., 2014). Thus, after comparing different models, the model of three related factors (Sense of existence, Freedom to give meaning to daily life, and Will to find meaning in the face of future challenges) proposed by Jonsén et al. (2010) is the one with the best fit in all countries. This finding suggests that, in the seven Latin American countries, the PIL is a multidimensional measure that is in line with the assumptions of Frankl’s existential theory, which involves the presence of three assumptions: freedom of will, the will to sense or meaning, and the meaning of life. The com bination of freedom of will and will to meaning generates the freedom a person has to pursue meaning in life. Likewise, the freedom to give meaning to life and the will to find meaning in future challenges involve creative values which are acquired through people’s experiences and attitudes (Frankl, 1958, 1963).

Unlike the original PIL model (Crumbaugh and Maholick, 1964), the Jonsén et al. (2010) model did not consider three items: (1) “When considering the world in relation to my life, the world: totally confuses me-Has meaning for my life,” (2) “I Am: hardly responsible-very responsible,” (3) “Regarding suicide: I have seriously thought it is a way out-I have never thought about it.” Regarding the first question, it has also been removed from the models reported in other studies (McGregor and Little, 1998; Morgan and Farsides, 2009; García-Alandete et al., 2013; Hayashi and Esmerelles, 2017). Apparently, this is a difficult question to answer due to the fact that it expresses the presence of a complex world to which it is not easy to make sense; while, in the second question, the difficulty may lie in the lack of knowledge and the need to see oneself as responsible (Jonsén et al., 2010). Finally, regarding the last question, it is always delicate to answer aspects associated with suicide (Desseilles et al., 2012), which would explain, in some way, its elimination from the model.

From a methodological point of view, the RMSEA values of the three-factor model are at the limit of what is typically considered as acceptable (Kline, 2015), as in the case of Mexico and Paraguay, or above, as in Argentina, Colombia, Ecuador, El Salvador, and Uruguay. At this point, it would have been possible to evaluate the presence of correlated errors among some of the items, which would have improved the model fit, and specifically the RMSEA value. The presence of correlated errors in factor models may suggest similarity in item content, higher task demands, measurement errors and response style (Brown, 2015). However, this assessment was not performed since it may overestimate or underestimate reliability due to the presence of variance that is not related to the construct (in this case, purpose in life) and generate a bias in the interpretation of measurement accuracy (Yang and Green, 2010).

Similarly, the reliability in the dimensions of the proposed three-factor model in most countries was higher than 0.65 (range 0.67 to 0.94), which is in a higher range than the range of 0.54 to 0.83 reported by Jonsén et al. (2010). However, in Paraguay the dimension “making sense of future challenges” had the lowest omega coefficient value (ω = 0.52), which was also reported previously (Jonsén et al., 2010). Therefore, it is suggested that the PIL is a relatively consistent measure of life purpose across samples. In the case of Paraguay, the relevance or not of using the third dimension would have to be evaluated, due to the low measurement precision.

Once the factor structure that best fit the data was determined, we proceeded to determine the MI among the seven countries. Applying the MGCFA method, no MI was reported (neither at the metric: ΔCFI = 0.04 or scalar level: ΔCFI = −0.02) of the PIL for the 7 countries involved, which would not allow for comparing the means between countries without absence of bias. As mentioned above, the classical MGCFA method for assessing MI presents some problems for total invariance, because possible equivalence violations increase as the number of groups to be compared increases, as is often the case for cross-national studies (Davidov et al., 2008, 2014; Muthén and Asparouhov, 2013; Asparouhov and Muthén, 2014). While it is possible to release parameters to achieve good fit and partial invariance, these modifications may produce an inadequate model that may be far from the real model (Asparouhov and Muthén, 2014). Releasing parameters does not guarantee that the means are unbiased; moreover, due to the multicollinearity present in the modification indices, the choice of released parameters could be arbitrary (Marsh et al., 2018).

Although there is a lack of evidence to support MI using the MGCFA method, invariance was also evaluated with the CFA-MIAL method (Marsh et al., 2018), which is suitable for comparing groups of countries, where MI is not expected due to cultural differences between them (Muthén and Asparouhov, 2018). In the present study, all factor weights are invariant, showing metric invariance; whereas 10.1% of intercept parameters are not invariant. Given that less than 25% of the non-invariant parameter estimates is the suggested cut-off point, it can be concluded that most of the PIL items showed scalar invariance. However, due to the novelty of the CFA-MIAL method, this criterion should be considered with caution until more evidence is obtained (Jang et al., 2017). Moreover, there is an absence of studies on the acceptable magnitude of non-invariance. Therefore, it is advisable to accumulate knowledge on the magnitude of non-invariance by reporting the estimates of factor loadings and intercepts, as well as their differences between groups, and to assess the impact of non-invariance in subsequent analyses (Kim et al., 2017). Having fulfilled the requirements based on a three-factor model for the PIL, the results of the more flexible CFA-MIAL method, based on realistic assumptions that relax invariance constraints, indicated that the factor structure of the PIL exhibits a pattern of approximate measurement invariance in the data (Asparouhov and Muthén, 2014). The analysis identified that the intersections of items 3, 4, 7, 10, 14, 15, and 20 show the highest degree of non-invariance. Thus, there seems to be variations in the way some indicators of life purpose are understood and interpreted in populations from different countries. This leads to the suggestion that these items may not be as robust in representing the life purpose of different populations and leads to the suggestion that possible comparisons should be interpreted with caution. On the other hand, the invariant items across the seven countries may be used as anchor or reference items when it is desired to construct a CFA model across countries. Achieving approximate invariance helps to estimate path coefficients and latent means, even if there are non-invariant items (Pokropek et al., 2019).

Although this is the first study on MI of PIL among samples from seven Latin American countries, it is important to recognize some limitations. First, the present study used non-probability convenience sampling. Therefore, the representativeness of the participants in each country is low, which limits the generalization of results to the entire general population of the participating countries. Second, although the total sample size is large (*N* = 4306), the selection was restricted to only 7 Latin American countries (Argentina, Colombia, Ecuador, El Salvador, Mexico, Paraguay, and Uruguay), which makes it even more difficult to generalize results beyond the case of Latin America. Third, it is also important to note the presence of an unequal representation of participants from Latin American countries. The current group of participants is dominated by samples from South American countries (Argentina, Colombia, Ecuador, Paraguay, and Uruguay) with little presence of participants from other regions of the Americas, such as Central America (El Salvador) and North America (Mexico). It is also important to carry out studies of this type in countries on other continents, such as Europe or Africa, in order to have more evidence of the universality or cultural relativism of the PIL. Fourth, it is important to consider the possibility that the data collection method overestimated the similarities between countries, since information was obtained from a population with similar characteristics, such as being Internet users and having experience in answering online surveys. However, some characteristics among the seven countries were not directly comparable at all, so comparisons of PIL scores may be somewhat biased. For example, Ecuadorians were under 25 years old on average compared to Uruguayans with an average age over 40 years old. With an average age difference of 15 years, the Uruguayans may be more predisposed by age to higher PIL scores. This has been shown previously, where people between the ages of 30 to 55 achieved significantly higher scores on the Spanish version of the PIL than younger people, between the ages of 18 to 29 (García-Alandete et al., 2013). Similarly, the samples from all countries were predominantly female (more than 60%) and the PIL findings may be more representative for women. Furthermore, the method of data collection did not allow for a cultural distinction between countries, as it did not obtain representative data on the subcultures within each country. Fifth, the study had a cross-sectional design, so a causal interpretation of the relationships between meaning in life and fear of COVID-19 cannot be established. Sixth, self-report measures were used to assess meaning in life and fear of COVID-19, which could lead to response bias due to social desirability and reduce the accuracy of the data. However, all participants were assured of their confidentiality and encouraged to answer all questions honestly. Seventh, although cross-cultural MI of the PIL was demonstrated, we were unable to examine why the intersections of some items were not invariant. It is possible that there are other potential predictors for the non-invariance of some items that were not considered in this study. It would be important to investigate factors contributing to life purpose and their effects on future behavior and life outcomes. Additionally, evidence of convergent or discriminant validity of the PIL with other constructs was not assessed. Finally, it is worth noting that the different factorial structures also reflect a marked deficiency in the specialized literature that does not allow for conceptual clarity of the dimensions that make up the PIL. In this sense, independently of investigating the metric properties, future studies should deepen efforts to reach a relative consensus on a more precise theoretical definition that clarifies the purpose in life construct and its facets.

Despite these limitations, this study has some important strengths to mention. First, this is the first study to evaluate the MI of PIL across different countries, specifically in this case, a large sample of seven Latin American countries. Second, the study is the first to employ the CFA-MIAL method to assess PIL invariance. As mentioned before, this is a novel statistical approach to perform joint invariance assessments in a large number of groups, in this case 7 countries. Third, beyond the general MI information, specific information on non-invariant items was provided. By identifying the non-invariant items, a better understanding of the individual PIL items is achieved. Fourth, the findings provide a strong contribution to increase the scope and application of the PIL in Latin America, which is particularly important due to the evidence that this scale is strongly related to aspects associated with well-being (Stoyles et al., 2015; Mei et al., 2021).

In conclusion, the results indicate that the PIL demonstrates MI in seven Latin American countries (Argentina, Colombia, Ecuador, El Salvador, Mexico, Paraguay, and Uruguay). This not only adds empirical evidence for the usefulness of the PIL in the Latin American context, but also contributes to the possibility of cultural comparison of life purpose using the same tool. Thus, important information on the similarity and or difference of life purpose between countries could be obtained. In addition, the understanding of cultural differences can be broadened by focusing on the identification of non-invariant items, non-invariant countries and sources of non-invariance. This is valuable in situations where cultural differences are apparently expected. Also, the results can guide researchers in developing culturally invariant items that measure aspects associated with life purpose. However, if the PIL is used in cross-cultural studies that measure and compare means of life purpose in different Latin American countries, it can be done with approximate invariance. This practice should be used with caution, as the impact of approximate invariance on factor means needs more research, and furthermore, there is still no consensus on whether approximate invariance is close enough to conclude that a measure is invariant (Jang et al., 2017).

## Data availability statement

The datasets presented in this study can be found in online repositories. The names of the repository/repositories and accession number(s) can be found in the article/Supplementary material.

## Ethics statement

The studies involving human participants were reviewed and approved by Ethics Committee of the Neuroscience Research Center of Rosario and the Laboratory of Cognition and Emotion, belonging to the Faculty of Psychology of the National University of Rosario in Argentina. The patients/participants provided their written informed consent to participate in this study.

## Author contributions

TC-R and LV provided initial conception, organization, and main writing of the text. LV and CL-C analyzed the data and prepared all figures and tables. MCe, MG, PM, MCa, CR, CL-C, RM-H, EC-A, ML-R, PC, YQ, ER, MP, OG, AB, MW, and CB-V were involved in data collection for their respective countries and acted as consultants and contributors to research design, data analysis, text writing, and read and approved the draft. All authors contributed to the article and approved the submitted version.
