# Supplementary material for: Cross-national measurement invariance of the Purpose in Life Test in seven Latin American countries
Source: Front Psychol. 2022 Sep 16;13:974133. doi: 10.3389/fpsyg.2022.974133 (PMC9524452; doi:10.3389/fpsyg.2022.974133)
Supplement: Supplementary file 1 [file Data_Sheet_1.PDF]

Supplementary material 1.

Descriptive analysis of the items by nationality of the participants

Country	Indices	Items																			
		1	2	3	4	5	6	7	8	9	10	11	12	13	14	15	16	17	18	19	20
Argentina (*n* = 1360)	M	4.6	3.9	5.8	5.8	3.9	5.4	2.4	5.4	5.8	2.5	5.4	4.6	6.1	3.9	4.1	5.7	2.4	2.9	3.1	5.7
	SD	1.5	1.8	1.4	1.5	1.8	1.5	1.8	1.3	1.3	1.8	1.7	1.9	1.2	1.9	2.1	1.8	1.5	1.6	1.5	1.3
	g1	-.5	.1	-1.5	-1.4	.1	-1.2	1.3	-1.1	-1.4	1.1	-.9	-.4	-1.5	-.1	.0	-1.2	1.1	.7	.7	-1.3
	g2	-.3	-1.1	1.8	1.5	-.9	1.1	.6	1.1	1.8	.2	.1	-.9	2.2	-1.1	-1.3	.2	.8	1.2	-.1	1.7
Colombia (*n* = 317)	M	4.7	1.7	6	5.9	3.8	5.5	2.2	5.4	5.8	2.6	5.4	5.1	5.9	3.6	3.8	5.5	2.4	2.7	3.1	5.6
	SD	1.4	1.7	1.2	1.4	1.6	1.5	1.6	1.3	1.3	1.7	1.7	1.7	1.3	1.9	1.9	1.8	1.5	1.6	1.5	1.3
	g1	-.5	.2	-1.6	-1.3	.1	-1.0	1.4	-.9	-1.1	.9	-.9	-.7	-1.5	.2	.2	-1.0	1.2	.9	.5	-1.2
	g2	-.5	-1.0	2.7	1.0	-.9	.7	1.3	.9	.5	-.1	.1	-.6	.3	-1.1	-1.2	-.1	.7	.3	-.3	1.5
Ecuador (*n* = 772)	M	4.5	4.1	5.9	5.8	4.1	5.5	2.4	5.3	5.7	2.9	5.2	4.8	5.7	3.4	4	5.6	2.7	2.9	3.3	5.8
	SD	1.4	1.8	1.2	1.4	1.8	1.6	1.8	1.3	1.4	1.8	1.7	1.8	1.3	2.0	2.0	1.7	1.7	1.8	1.6	1.3
	g1	-.3	-.1	-1.3	-1.2	-.1	-.9	1.3	-.7	-1.0	.7	-.8	-.4	-1.1	.4	-.0	-.9	.9	.8	.6	-1.2
	g2	-.2	-.9	1.3	.9	-.9	.4	.8	.3	.3	-.6	-.3	-.8	1.1	-1.1	1.2	-.2	-.0	-.2	.1	1.0
El Salvador (*n* = 309)	M	4.8	3.9	6.3	6.2	3.8	5.7	2.3	5.6	6.0	2.6	5.8	5.5	6.2	3	3.6	6.0	2.3	2.5	2.9	6.2
	SD	1.5	2.0	1.1	1.3	2	1.5	1.8	1.4	1.3	1.9	1.6	1.7	1.1	2.1	2.2	1.6	1.7	1.7	1.7	1.2
	g1	-.4	.1	-2.2	-1.8	.1	-1.3	1.4	-.9	-1.5	.9	-1.5	-.9	-1.8	.7	.3	-1.6	1.5	1.2	.7	-1.8
	g2	-.4	-1.2	5.1	2.6	-1.2	1.3	1.1	.5	1.7	-.1	1.5	.1	3.9	-.9	-1.3	1.7	1.4	.5	-.4	3.4
Mexico (*n* = 904)	M	4.9	3.6	6.0	6.2	3.8	5.9	2.9	5.6	6.1	2.4	5.8	5.3	5.9	2.9	3.7	5.9	2.4	2.6	2.7	6.0
	SD	1.4	1.8	1.3	1.2	1.8	1.4	2.1	1.3	1.2	1.7	1.5	1.8	1.2	1.9	2.1	1.6	1.6	1.6	1.5	1.2
	g1	-.6	.3	-1.6	-1.8	.2	-1.4	.8	-.9	-1.5	1.2	-1.3	-.9	-1.4	.7	.3	-1.6	1.3	1.2	.9	-1.5
	g2	-.2	-1.0	2.3	3.0	-.9	1.9	-.8	.8	2.1	.5	1.1	-.3	1.9	-.7	-1.2	1.5	1.0	.7	.4	2.3
Paraguay (n = 244)	M	4.8	3.9	6.0	5.9	4.1	5.6	3.0	5.6	5.9	2.4	5.6	5.3	5.9	3.2	3.7	5.7	2.3	2.8	3	5.9
	SD	1.4	1.8	1.3	1.4	1.8	1.5	2.2	1.3	1.2	1.7	1.7	1.8	1.4	1.9	2.1	1.7	1.5	1.7	1.6	1.2
	g1	-.6	.1	-1.8	-1.3	-.1	-1.2	.7	-1.1	-1.3	1.1	-1.2	-.8	-1.5	.5	.1	-1.1	1.3	.9	.7	-1.2
	g2	-.3	-1.0	3.7	.8	-1.0	.9	-.9	1.4	1.8	.2	.5	-.5	2.1	-1.0	-1.2	.1	1.4	-.2	-.2	1.3
Uruguay	M	5.0	3.5	5.9	6.1	3.8	5.6	2.3	5.6	6.1	2.2	5.9	5.4	6.3	3.7	3.8	6.0	2.3	2.6	2.7	5.9

Country	Indices	Items																			
		1	2	3	4	5	6	7	8	9	10	11	12	13	14	15	16	17	18	19	20
(n = 400)	SD	1.3	1.6	1.3	1.4	1.5	1.3	1.6	1.2	1.2	1.5	1.4	1.7	1.1	1.7	1.9	1.5	1.4	1.3	1.4	1.2
	g1	-.9	.4	-1.6	-2.0	.2	-1.1	1.3	-1.3	-1.6	1.4	-1.4	-.9	-1.9	.2	.1	-1.5	1.4	.9	.9	-1.3
	g2	.9	-.7	2.7	4.1	-.6	1.9	1.0	2.3	2.8	1.2	1.7	-.1	4.7	-.9	-1.1	1.0	1.8	.6	.7	1.7

Note. *M*=Mean; *SD*=Standard Deviation; *g1*= Skewness; *g2*= Kurtosis
